# Modular dual mobility articulation in primary and revision hip arthroplasty: lights and shadows

**DOI:** 10.1186/s13018-023-03730-8

**Published:** 2023-04-05

**Authors:** Alessandro Moghnie, Domenico Tigani, Alberto Consoli, Emanuela Castiello, Marco Ganci, Luca Amendola

**Affiliations:** Department of Orthopaedic Surgery, Ospedale Maggiore C.A. Pizzardi, Largo B. Nigrisoli 2, 40133 Bologna, Italy

**Keywords:** Modular dual mobility cup, Jumping distance, Total hip arthroplasty, Dislocation

## Abstract

**Purpose:**

The use of dual mobility cups in total hip arthroplasty has gained popularity in light of the fact it enables to reduce dislocation through increased jumping distance (JD) and impingement-free arc of movement. Modular Dual Mobility Cup (modular DMC) systems have been recently introduced to enable the use of dual mobility cups with standard metal-backed shells. The objective of this study was twofold: calculate the JD for each modular DMC system and conduct a systematic literature review to report clinical outcomes and reasons for failure of this construct.

**Methods:**

The JD was calculated using the Sariali formula: JD = 2*R*sin [(*π*/2 − Ψ − arcsin (offset/*R*))/2]. A qualitative systematic literature review was conducted according to the Preferred Reporting Items for Systematic Reviews and Meta-Analyses guidelines. A comprehensive search for English and French articles between January 2000 and July 2020 was run on PubMed, EMBASE, Google Scholar, and Scopus with the primary objective of finding articles about modular DMC systems.

**Results:**

We identified eight 8 different manufacturers of modular DMC systems and 327 publications on the subject. After screening for duplicates and eligibility, we identified 229 publications: 206 articles were excluded because they contained no reports on modular DMC systems, whereas other three were not included because they focused on biomechanical aspects. Among the 11 included articles, 2 were prospective case series, 9 were retrospective case series. True dislocation occurred in 25 cases (0.9%), and six of them were solved by closed reduction without necessity of revision, while all 5 intraprosthetic dislocations were operated.

**Conclusions:**

Modular DMCs are a valid method to deal with complex THA instability, with good clinical and patient-reported outcomes, low complication rates, and low revision rates at early follow-up. We would advise cautious optimism on the role of modular DMC implants, as it seems safer to use ceramic instead of metallic heads whenever possible to avoid the increase cobalt and chromium trace ion serum levels.

## Introduction

Dislocation following Total Hip Arthroplasty (THA) remains the leading reason for revision in the first 2 years after surgery [1]. Approximately 50% of dislocations occur within the first 3 months after the index procedure, and more than 75% occur within the first year [2].

The risk of THA dislocation increases due to multiple causes. Risk factors are traditionally divided in patient-related and technical-related [3].

The first group of factors includes diagnosis (OA or FNF for example), age, gender, comorbidities, neuromuscular diseases, etc.; the second includes surgical approach, soft tissue management, implant design choice, and component positioning [4].

A prospective case–control study was recently carried out on a large population in France [5]. The authors were able to correlate the risk factors of instability with the following features: high ASA score, neurological disability, history of spinal disease (lumbar stenosis, spinal fusion, discectomy, scoliosis and injury sequelae), unrepaired joint capsule (especially when using the posterior approach), and cup inclination outside Lewinnek’s safe zone.

They found that if the capsule is not repaired, the dislocation rate increases by six times when the posterior approach was used and by four times when the anterior approach was used.

Since it was developed in the late seventies, the dual mobility (DM) cup has shown to be a good solution to this problem [6–8], leading to a significant reduction in the instability rate. These reported clinical data have recently been confirmed by European Registers [9–12]. In France, the country where the DM cup was developed, the dislocation rate has globally decreased from 9.06% in 2005 to 6.10% in 2014 [13] and dual mobility constructs are nowadays estimated to be used in 62% of all revision hip procedures [14] with substantial cost savings [15].

Two options for Dual Mobility implants are currently available: monoblock or modular [16].

The monoblock design consists of a one-piece steel or cobalt chrome acetabular shell. Some implants also have two pegs at the bottom (in correspondence of the pubis and ischium) and one screw on the top of the cup. Furthermore, a hook for obturator anchorage and one or more plates can be used with some implants for ilium screw fixation.

The monoblock component does not permit screw fixation through the shell. This option is usually preferred in revision settings.

Modular Dual Mobility Cup (modular DMC) cups were introduced during the last decade in order to overcome this limitation.

They enable screw fixation of the outer titanium shell and have a high polished cobalt-chromium liner which is inserted and press-fixed via a taper junction in order to isolate the titanium from the polyethylene liner.

Early outcomes are encouraging, but the additional bearing surface reduces the effectiveness of the large head, increasing the potentiality of metal corrosion and PE wear; moreover, there is the risk of intra-prosthetic dislocation recurrence.

The purpose of this study was to:

1. analyze all the Modular Dual Mobility implants present on the market and calculate the jump distance (JD) of each implant
2. compare the jump distance of modular DMC implants with that of monoblock DM implants combined with standard cups of different head diameters
3. evaluate the dislocation rate and intraprosthetic dislocation rate for each implant
4. assess the complication rate of modular DMC

## Materials and methods

We conducted a qualitative systematic review of the literature according to the Preferred Reporting Items for Systematic Reviews and Meta-Analyses guidelines [17] in order to analyze the results of primary and revision hip arthroplasty. Literary research was performed on all published and available reports in global databases. In particular, we used available data from the following databases: PubMed, EMBASE, Google Scholar. Ethics approval was not required because patients were not involved in the conception, design, analysis, drafting, interpretation or revision of this research. The inclusion criteria take into account all studies on primary hip arthroplasty and hip revisions in which a modular DMC prosthesis was implanted. Biomechanical studies, technical notes, letters to editors, case reports, and expert opinions were excluded. Reference lists of the included articles were carefully checked for missed studies. Both English and French studies were evaluated. Key words used in the search strategy included “modular dual mobility” and “modular dual-mobility.” All the prostheses that partially or completely matched these criteria were selected. We included all the articles published from January 2000 to July 2020. Two independent reviewers (A.M. and A.C.) with experience in international literature screened the titles and abstracts of all the articles in order to evaluate their inclusion in, or exclusion from, the study. The search analysis also involved the consultation of various Internet databases of current and past DM implant manufacturers.

The primary outcome of the literature analysis conducted on published articles was to consider the incidence of postoperative dislocation after primary or revision THA requiring closed reduction, open reduction, or revision. Secondary outcomes included rates of intraprosthetic dislocations (IPD) and related complications reported in the literature.

The jump distance was calculated for standard hip arthroplasty, monoblock DM implants, and for each modular DMC captured according to the formula created by Sariali et al. [18]

Considering the 2 possible rotations of the cup, they calculated these values:

- value *α*: the cup frontal abduction angle, which rotates around the cranio-caudal axis
- value *β*: the cup anteversion angle, which rotates around the antero-posterior axis

The planar cup inclination angle (Ψ) measured through X-rays can be calculated based on these values with the following formula:

1 $$\Psi = \arctan \left[ {\tan \left( \alpha \right) \times \cos \left( \beta \right)} \right].$$

Considering the offset of the femoral head, defined as the distance between the femoral head center and the cup opening plane (Fig. 3), they considered these variables:

- femoral inset: if the femoral center was located inside the cup, and the offset was negative
- femoral offset: if the femoral center was located outside the cup, and the offset was positive

The jumping distance can be calculated using definitive formula:

$${\text{JD}} = 2R\sin \left[ {\frac{{\left( {\pi /2} \right) - \Psi - \arcsin \left( {{\text{offset}}/R} \right)}}{2}} \right].$$

We considered the value of alfa and beta angles as constant to eliminate confusing factors related to acetabulum positioning and to focus exclusively on evaluating offset impact on acetabular stability and obtained a JD value based on the offset values reported by each company (Table 1).

**Table 1:** Jump distance of different head sizes in standard cups

	Head 22	Head 28	Head 32	Head 36
*Data*				
Alfa	45.00°	45.00°	45.00°	45.00°
Beta	15.00°	15.00°	15.00°	15.00°
Offset	0 mm	0 mm	0 mm	0 mm
*R*	11 mm	14 mm	16 mm	18 mm
*Intermediate*				
tan(*α*)	1	1	1	1
cos(*β*)	0.97	0.97	0.97	0.97
tan(Ψ) = tan(*α*) * cos(*β*)	0.97	0.97	0.97	0.97
Ψ = arctan(Ψ)	44.01°	44.01°	44.01°	44.01°
ϴ = arcsin (offset/*R*)	0.00°	0.00°	0.00°	0.00°
*Results*				
JD = 2*R* sin((*π*/2 − Ψ − ϴ)/2)	8.6 mm	10.9 mm	12.5 mm	14.1 mm

## Results

**Fig. 1:**
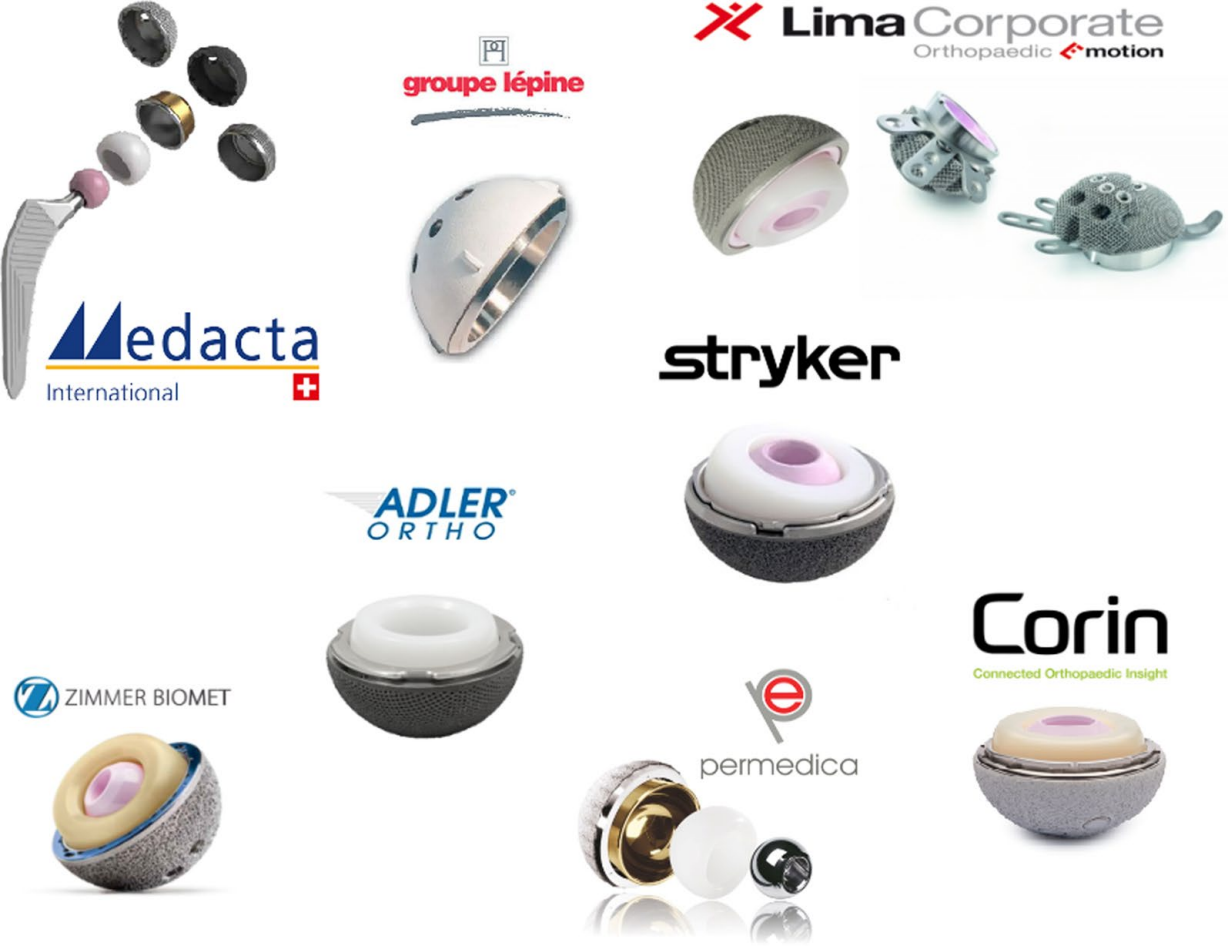
Implants identified

Our research led us to identify 8 different manufacturers of acetabular constructs with the aforementioned characteristics of modularity, three of which manufacture modular DMCs for more than one implant (Fig. 1). All groups are discussed in this article and summarize in Fig. 1.

### Variation of JD and head diameter

A different jump distance (JD) was calculated for each modular DMC based on constant values for alfa and beta angles (cup inclination and anteversion), and offset data were obtained from each manufacturer.

A standard cup with a head of 22, 28, 32 and 36 mm (Table 1) was considered.

**Fig. 2:**
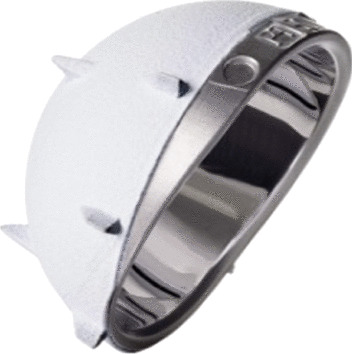
Groupe Lépine, Quattro cup

A single monoblock, non-modular DMC was taken as reference comparator (Groupe Lépine, Quattro cup) (Table 2, Fig. 2).

**Table 2:** Jump distance of Quattro cup, Groupe Lépine

	Quattro 54–48
*Data*	
Alfa	45.00°
Beta	15.00°
Offset	1.45
*R*	24 mm
*Intermediate*	
tan(*α*)	1
cos(*β*)	0.97
tan(Ψ) = tan(*α*) * cos(β)	0.97
Ψ = arctan(Ψ)	44.01°
ϴ = arcsin (offset/*R*)	1.73°
*Results*	
JD = 2*R* sin((*π*/2 − Ψ − ϴ)/2)	17.4 mm

In the group of standard cups, the minimal value of 8.6 mm was obtained using the 22.2 mm head, while the JD reached a value of 14.1 mm when a 36 mm head was used (Table 1).

The highest JD value, 17.4 mm, was achieved with the monoblock DM system used as reference comparator.

Monoblock DM cups present a 6 mm nominal size difference between the head and shell; therefore, for the studied acetabular size of 54 mm, the actual size of the polyethylene liner is 48 mm. A greater variability was observed in the modular group because of a difference in terms of manufacturer features. Integra acetabular shells (Groupe Lépine) and Stryker’s MDM® system with Trident PSL cup have the highest JD value of all modular constructs (16.6 and 15.7 mm). The first implant, which is only available in 4 sizes ranging from 50 to 62, has a 46 mm polyethylene insert for the 54 mm size; it therefore offers a nominal size difference between the head and shell of 8 mm. The same difference is possible for Stryker’s MDM® system, as long as the PSL Trident shell is used. As for the other two constructs, where the MDM® Trident and Tritanium hemispherical shells can be used, the nominal size difference reached 12 mm, so the liner size for a 54 mm acetabular implant is 42 mm. The same value was observed for Adler Ortho and Medacta International implants, whereas the G7 Zimmer Biomet construct uses a 44 mm liner for the 54 cup with a difference of 10 mm.

The lowest JD value (13.8 mm) was produced by Corin Trinity. This company chose to only provide two liners—one of 40 mm and another of 42.5 mm—regardless of the size of the shell. The Lima company opted for a similar solution for the del Delta one and Delta Revisione implants, which only accept 40 mm and 42 mm liners. Calculating JD for these implants was not possible due to the absence of the offset value (Table 3).

**Table 3:** Jump distance of the implants considered

							
	Trinity 54–40	Trinity 54–42.5	Permedica 54–40	Tritanium hemispherical shell 54–42	Trident PSL shell 54–46	Trident hemispherical shell 54–42	G7 54–44	FIXA TI-POR bis mobility 54–42	Integra 54–46	Medacta 54–42
*R*	20 mm	21.5 mm	20 mm	21 mm	23 mm	21 mm	22 mm	21 mm	23 mm	21 mm
JD	13.8 mm	14.8 mm	14.5 mm	14.2 mm	15.7 mm	14.2 mm	17.2 mm	13.6 mm	16.6 mm	14.3 mm

### Study selection

A total of 327 papers were captured through the literature search.

After screening for duplicates and eligibility, we identified 229 publications.

A total of 207 articles were excluded because they reported no modular DMCs.

Of the 22 full text articles assessed for eligibility, 3 were excluded because they were biomechanical or science studies, while other 8 were excluded because they only took into account serum metal levels, and not the outcome we were interested in.

**Fig. 3:**
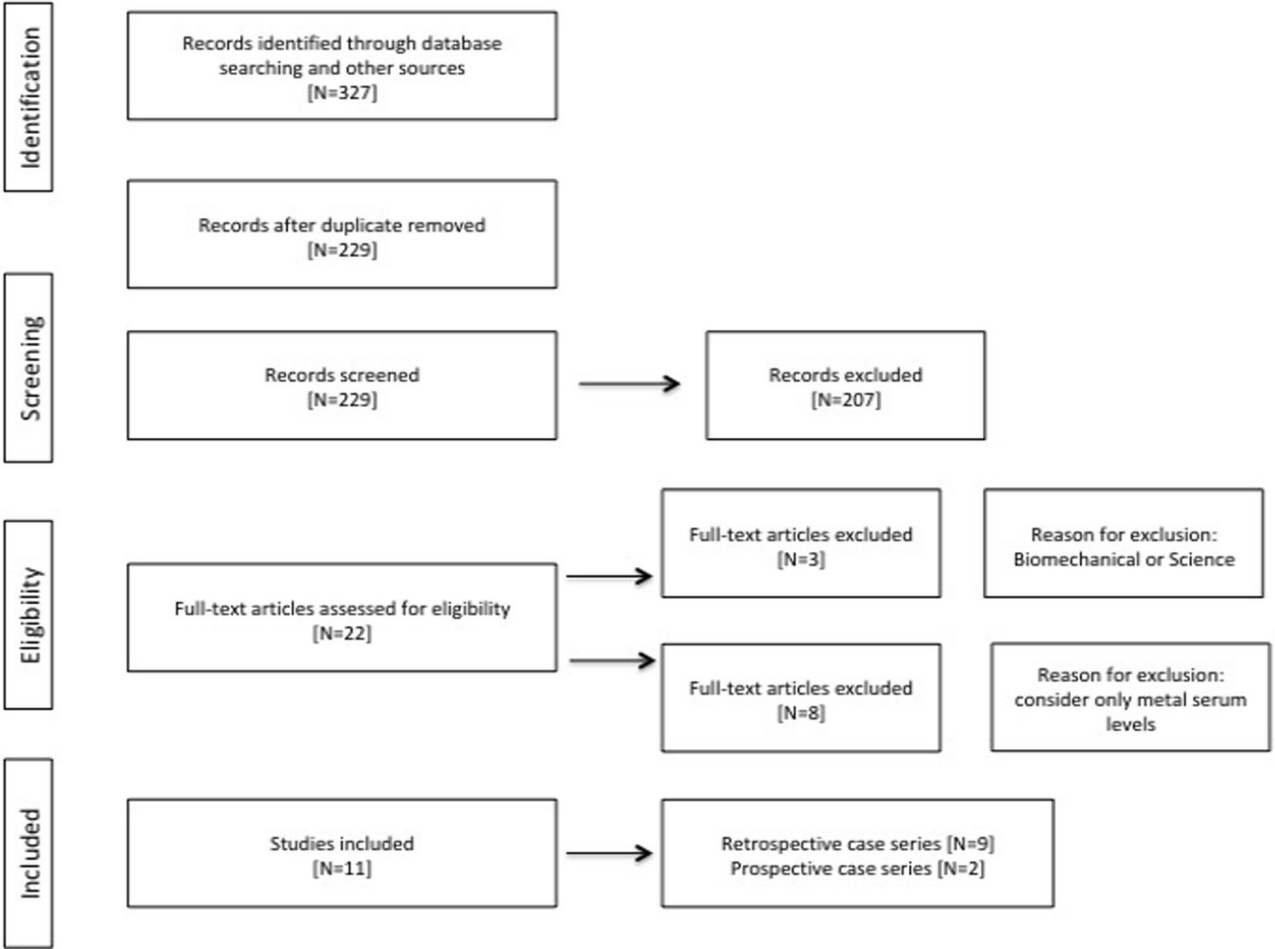
Articles flow chart

Among the 11 included articles, 2 were prospective case series, 9 were retrospective case series. [19–31] (Fig. 3).

Two of them only focused on malseating of metal liner, the rest considered dislocation, instability and IPD [21, 24].

A total of 2001 implants were considered, of which 1330 were primary hips and 671 revisions. A total of 1854 patients received MDM Stryker acetabular cementless prostheses using Trident constructs or Tritanium implants [24–27, 29–31]. Two of the articles reported the use of Groupe Lépine’s Integra implants in 59 patients [19, 20]. One article reported the results of 88 G7 Zimmer implants [28]. We were not able to find any papers on the use of Delta TT System (Lima Corporate Spa), Trinity (Corin), Bismobility (Adler), Traser (Permedica) and Medacta implants at the time of the literature research.

We included data on the clinical evolution of the implanted cups in order to evaluate modular DMC outcomes and specific complications. Dislocation occurred in 25 cases on 2001 (1.25%); 7 of which were solved by means of closed reduction without necessity of revision, which was, on the contrary, necessary in 18 hips. True dislocation occurred in 22 of 671 revision cases (3.27%) and in 3 of 1330 primary cases (0.23%). Early IPD occurred in 4 patients after closed reduction due to a “bottle opener” effect [32]. Another patient proceeded with hip dislocation self-reduction causing IPD. All 5 patients need surgical treatment.

Two cases of IPD occurred within the series of 66 MDM Stryker cups, whereas three of the cases occurred within the series of 88 G7 Zimmer cups.

Improper seating of the metallic liner was observed in 37 hips, as reported in three papers; all cases concerned MDM constructs. More specifically, this complication presented with 9 Trident I shells, 9 Trident II shells and 8 Trident I PSL shells [30]; the shells that had been used in the remaining 11 cases were not specified [24, 31, 32].

All the malseating were treated with revision of the only liner.

## Discussion

We were able to identify 7 different modular DMC construct manufacturers, three of which provide the possibility to use the same polyethylene liner for different designs. Implant characteristics were studied for each shell, and the JD was calculated based on a 54 mm size cup according to formula described by Sariali et al. [15]. The reference comparator we used was the monoblock DM implant, which has a 6 mm reduction in the outer diameter of the liner depending on the size of the implant and a JD value of 17.4 mm with the reference cup size of 54 mm. All modular constructs have a JD value reduction due to the presence of a modular CoCr alloy liner with variable decrease in the inner diameter of the cup. Tigani et al. have recently calculated how jumping distance (JD) and CR lateralization change when considering a conventional DM cup, a modular DMC and an FB cup by using an analytical 3D-modelling simulation [33]. They matched for same cup size, of a single company, according to cup abduction, anteversion angles, head diameters and femoral head offset. With DM cups, the JD linearly increased as cup size increased, whereas with the modular implant, the JD slightly increased up to the 56 mm cup size and then, remained approximately constant at 15.1 mm. These Authors highlight that the JD does not only depend on femoral head size and cup positioning—more specifically on the abduction angle and anteversion angle [18]—but also on femoral offset. Head offset can have a positive or negative value (inset). Therefore, according to data from Tigani et al. [33] offset progressively increases with cup size in modular DMC implants, and JD remains low if compared with monoblock DM implants. In the latter, offset progressively decreased but always remained negative (inset), thus leading to an increase in the JD. The Authors are cautions in considering these results were valid for all implants, since they cannot exclude variability with others devices.

The difference in liner diameter between the modular DMC systems and corresponding monoblock DM cups tends to remain constant even for smaller implants, without ever falling below the threshold of 32 mm for size 42 G7 Zimmer and Adlerortho BIS MOBILITY implants and size 36 Stryker MDM and Traser Permedica implants. Groupe Lépine does not produce modular acetabular shells under the size of 50 mm for the Integra cup, so the smallest liner is 38 mm and requires a 22.2 mm head. Lima Corporate only produces 40 and 42 mm liners, which can both be implanted according to the characteristic of the modularity. The same philosophy has been adopted by Corin, whose only liner sizes are 40 mm and 42.5 mm.

In our review, modular DMCs led to very low rates of instability and necessity of reoperation in both primary and revision cases, amounting to only 25 cases of the 2001 total implants (1.25%). Twenty-two dislocations occurred in the revision setting, while only three involved primary cases.

In addition to one study considering both primary and revision THA, the number of studies focusing on primary THA is comparable to the number of studies focusing on revision THA (4 vs. 6). However, primary implants are twice as many as revisions (1330 vs. 671), so the system can be said to effectively reduce the risk of dislocation in both situations.

We can estimate an incidence rate of post-operative dislocations of 1.25% in 2001 reported implants (0.23% occurred in primary procedures (3 in 1330), whereas 3.28% involved complicated construct revisions (22 in 671). However, the incidence of surgical revisions was even lower as 7 in 25 cases benefitted from closed reduction. Overall, these outcomes were comparable with other studies on monoblock implants for both primary and cemented revision cases [9–12]. In the largest multicenter study of this review, Huang et al. [24] observed that recurrent instability was associated with an outer diameter of the polyethylene insert inferior to 38 mm. In fact, DM liners that are 38 mm or smaller had a significantly higher rate of dislocation (16%) compared to 42 mm or greater liners (3.5%; P ¼ .03). No other studies confirmed the same results. Addona [28] reported an even higher incidence with bigger liners; dislocation occurred in 3 cases out of a total of 56 liners of less than 40 mm (1.8%) versus 4 dislocations out of a total of 98 liners sized over 40 mm (2.4%).

The same Authors reported 5 cases of early intraprosthetic dislocation (IPD) that occurred while attempting a closed reduction on 7 dislocations. IPD occurred in 2 out of 3 Stryker constructs and 3 out of 4 G7 systems. These data align with the 2017 review by De Martino et al. [31], who emphasized the high likelihood of iatrogenic etiology for early IPD, whereby the dissociation of the head and liner occurs when attempting a closed reduction in a large joint dislocation.

Despite encouraging results in terms of instability and the significant increase in the application of this technology, concerns on modularity have been raised due to the potential malseating of the liner or fretting corrosion at modular junctions [30, 31, 34]. The notion that a stiff cobalt-chrome liner exposes to a potentially higher risk of malseating because of a lower conforming tolerance than a polyethylene liner is supported by experience with incomplete liner seating with metal-backed ceramic liners [35, 36]. Causes of malseating include the interposition of soft tissue or bone and plastic deformation of the acetabular shell during impaction. Cadaveric studies using the press-fit technique with Trident acetabular shells have actually shown that constant compression deformation prevents complete liner seating [6, 35]. A number of cases of malseating with modular DMCs were reported in two papers [35, 36], both considering Stryker’s MDM construct. In a cohort of patients who underwent modular DMC implantation performed by 17 different surgeons using the MDM [35] liner, the reported incidence of malseating of the metallic liner reached a value of 5.8%, i.e., 32 of 551 MDM liners [35]. The incidence of malseating was significantly higher among low-volume MDM surgeons than among high-volume MDM surgeons. Malseating of the liner was observed in all three different cup designs from Stryker: Trident I, Trident II (both hemispherical) and the Trident peripheral self-locking rim flare design. Causes of malseating included interposition of soft tissue or bone and plastic deformation of the acetabular shell during impaction. Prior cadaveric studies using the press fit technique with Trident acetabular shells have actually shown that constant compression deformation prevents complete liner seating [35].

In 2020, Chalmers reported about four (1.3%) patients (4 THAs) presenting radiographic evidence of incomplete liner seating with prominence at the inferior acetabular rim in a cohort of 305 hips [36].

Even though this rate of liner malseating is significantly lower than those in reports of similarly hard and inelastic metal-backed ceramic liners, the Authors warn about the need to verify linear seating circumferentially, especially to test the inferior part of the liner, since that is the most challenging location for the surgeon to visualize intraoperatively and it is also the location where all the liners were incompletely engaged.

This review reveals several limitations. First of all, none of the studies included were randomized controlled trials: most were retrospective case series, and only two were prospective. Moreover, the methodological quality has to be considered low because the series. Nonetheless, the low incidence of complications in a population at such a high risk of instability could help understand the real advantage of modular cups.

The large head has shown to contribute to stability as well as to a potential increase in impingement-free range of motion due to second articulation and, ultimately, to the real increment of the jump distance. The real increment of the jump distance is actually inferior with the modular design despite the fact the monoblock DMC offers a 6 mm nominal size difference between the head and shell. With the modular design, the head-to-cup difference could be even more than the double; it depends on the type of implant, and such difference could be present even within the same company. Some reports warn about a possible higher incidence of dislocations when using liners that are smaller than 38 mm; although these data have not been confirmed by other works, we must note that at least half of the companies do not produce modular DMC systems with smaller sizes than 50 or 52 mm.

An intraprosthetic dislocation (IPD) occurs when the inner femoral head is separated from the outer polyethylene liner. In their review, De Martino et al. [32] distinguished between early and late IPD: IPD is defined as early when it occurs within 24 months. Late IPD, which occurs after 24 months, is mainly related to retentive rim wear, as observed by Lecuire et al. [37], which leads to the failure of the capture mechanism between the mobile polyethylene liner and the femoral head. This complication depends on the head/neck ratio as well as on the shape and roughness of the neck involved [4]. Femoral necks with an unpolished surface and large diameters should be avoided. For similar reasons, care should be taken to ensure that the base of the Morse taper is fully covered by the femoral head, avoiding skirted femoral heads that can cause impingement with the polyethylene liner. The application of these rules has contributed to an almost complete disappearance of this complication with the latest generation of monoblock dual mobility cups [38].

Early IPD is rarely described in European series—the first one to be reported was in 2011. Early IPD can be considered as the mechanical failure of the retentive rim without a wearing process. According to De Martino [40], a iatrogenic etiology is highly probable, since in most cases the dissociation of the head and liner occurs when attempting the closed reduction in a large joint dislocation. As described by Aslanian [5], liner features of the different DMCs depend on four main factors: first, the diameter and relative position of the retentive ring to limit any harmful contact with the femoral stem; second, the over-covering surface of the head to create an intraprosthetic jump distance; third, the presence of protective beveled edges (chamfers) in contact areas with the prosthetic neck; and fourth, appropriate polyethylene elasticity in order to allow the passage of the head through the retention rim, avoiding the plastic deformation of the latter, which would otherwise lead to the failure of the capture mechanism. For this reason, it would be helpful if manufacturers provided these technical data. Anyway, in the setting of large joint dislocations, precautions should be taken during closed reduction to prevent iatrogenic IPD, including adequate muscle relaxation and fluoroscopic guidance [32].

An outer diameter of the polyethylene liner inferior to 38 mm, a 22 mm (vs 28 mm) inner head size, and an isolated liner exchange to modular DMC with retention of the acetabular component were risk factors for recurrent dislocation in literature. It is worth to note that the 36 mm and 38 mm liners are the smallest liners available in the modular DMC system; they are sizes C and D, respectively, and both mate with a 22 mm head.

The modular version enables surgeons to use this construct in complex primary or revision surgery, where the use of screws is desirable.

Modularity potentially entails additional complications such as liner malseating and elevation of metal ions.

## Conclusions

In conclusion, modular DMCs are a valid method to deal with complex THA instability. Our study suggests good clinical and patient-reported outcomes, low complication rates, and low revision rates at early follow-up. Even though at least eight companies have developed this type of implants, at present most data are correlated with Stryker's MDM liner, which mates with both Trident (II and SPL) and Tritanium shells; two papers report Lépine’s Integra modular implant, whereas only one paper focuses on the G7 Zimmer implant. We would advise cautious optimism on the role of modular DMC implants as some concerns still remain regarding the use of this solution in patients with high cobalt and chromium trace ion serum levels, such as those observed in some of the cases submitted to revision for MoM-related complications. Beyond these concerns, there do not seem to be any worrying data on possible reactions in well-functioning implants. However, it seems safer to use ceramic instead of metallic heads whenever possible.
